# Sodium Danshensu Cream Promotes the Healing of Pressure Ulcers in Mice through the Nrf2/HO-1 and NF-κB Pathways

**DOI:** 10.3390/ph15121548

**Published:** 2022-12-13

**Authors:** Fei Yang, Cuizhen Shen

**Affiliations:** 1 School of Nursing, Zhejiang Chinese Medical University, Hangzhou 310053, China; 2 Hangzhou Women’s Hospital (Hangzhou Maternity and Child Health Care Hospital), Hangzhou 310008, China

**Keywords:** pressure ulcers, sodium Danshensu cream, ischemia/reperfusion injury, Nrf2/HO-1 pathway, NF-κB pathway

## Abstract

On the basis of the mice pressure ulcers (PU) model, the protective effect and potential mechanism of sodium Danshensu (SDSS) cream against PU were investigated. The mice were randomly divided into three groups: the negative control group (cream without 0.5 g SDSS), the SDSS group (cream containing 0.5 g SDSS), and the positive group (0.5 g Hirudoid^®^). After 7 and 14 days of ointment application, the wound-healing rate of the SDSS and positive groups was significantly higher than that of the control group (*p* < 0.05). The results of hematoxylin–eosin staining also indicated that SDSS has the potential to promote the healing of PU. In addition, the serum IL-6, IL-1β, TNF-α, and MDA levels decreased significantly (*p* < 0.01) after 14 days of SDSS treatment, while the SOD, CAT, and GSH-Px activities increased significantly (*p* < 0.01). In addition, SDSS cream was able to significantly increase the expression of Nrf2, HO-1, GCLM, NQO1, NF-κB p65, NF-κB p50, IKKα, and IKKβ while decreasing the expression of Keap1 and IκBαin the Nrf2/HO-1 and NF-κB pathways. Our research will provide a foundation for the future clinical prevention and treatment of PU with SDSS cream.

## 1. Introduction

Pressure ulcers (PU), also known as pressure injuries or pressure sores, are one of the five major factors that adversely affect patients’ quality of life and are also one of the most expensive complications [1,2]. Numerous critical and persistent diseases are associated with PU [3,4]. In countries with a high standard of living, such as the United States, Germany, and Australia, the prevalence of PU ranges from 7 to 14% [5,6]. In developing nations with a median income, such as Brazil and Indonesia, the prevalence of PU ranges from 8% to 66% [7,8]. However, the occurrence of PU has serious consequences for patients, their families, and the medical staff involved, and it increases the workload of caregivers and nursing staff. In addition, the incidence of PU increases the length of patients’ hospital stays and their medical expenses [9]. According to research, the annual cost of treating PU is very high in many countries, including the United States, which spends $17.8 billion annually on PU treatment [10]. Therefore, the appropriate and effective prevention and treatment of PU is a crucial concern for both patients and medical staff.

Ischemia/reperfusion (I/R) injury refers to the pathological process that exacerbates tissue and organ injury when blood flow is restored after a prolonged period of ischemia [11,12]. Several tissues and organs of the body are susceptible to I/R injury, including the liver, brain, myocardium, kidney, gastrointestinal tract, and skin [13,14,15,16]. Consequently, how to prevent and treat I/R injury in ischemic tissues or organs has become a current hot topic. It is believed that the mechanism of I/R injury is mainly associated with energy deficiency, excessive release of oxygen free radicals, excessive activation of leukocytes, calcium overload, activation of inflammatory cytokines, apoptosis, etc. [17,18,19]. Oxygen free radicals and inflammatory responses are among the research hotspots [20,21,22]. In addition, the Nrf2/HO-1 and NF-κB pathways are two of the most commonly used pathways to explain oxygen free radicals and inflammatory responses [23,24]. For example, Morsy et al. [23] discovered that paeonol could attenuate hepatic I/R injury by modulating the Nrf2/HO-1 and TLR4/MYD88/NF-kappa B pathways. Wang et al. [24] found that curcumin could protect against hepatic I/R injury through inhibiting the TLR4/NF-kappa B pathway. 

Danshen (*Radix Salviae Miltiorrhizae*) is one of the most commonly used traditional Chinese medicines [25,26]. Danshensu (DSS) is a water-soluble active ingredient in Dan Shen, but it is inherently unstable [27]. As the sodium salt of DSS, sodium Danshensu (SDSS) possesses stable properties as well as high absorption and utilization [28,29]. Recently, SDSS has been utilized to reduce I/R injuries in a variety of tissues [30,31,32]. By inhibiting apoptosis, Guo et al. [30] demonstrated that SDSS has a neuroprotective effect against cerebral I/R injury in rats. Gao et al. [31] demonstrated that SDSS can reduce cerebral I/R injury by targeting the AKT1 protein. In previous research, we discovered that SDSS has a protective effect in promoting the healing of stage 2 pressure injury wounds in rats subjected to I/R injury [32]. In our previous studies, SDSS was administered intraperitoneally, which undoubtedly increased the pain of patients, and its molecular mechanism to promote wound healing has not been thoroughly investigated. In this study, SDSS cream was first prepared, and its effect on promoting the healing of PU in the model of stage 2 PU in mice was observed. In addition, the Nrf2/HO-1 and NF-κB pathways were selected to evaluate the potential protective mechanism against PU. Our findings will serve as a theoretical and experimental foundation for the clinical prevention and treatment of PU with SDSS.

## 2. Results and Discussion

### 2.1. Establishment and Evaluation of PU in Mice

**Figure 1:**
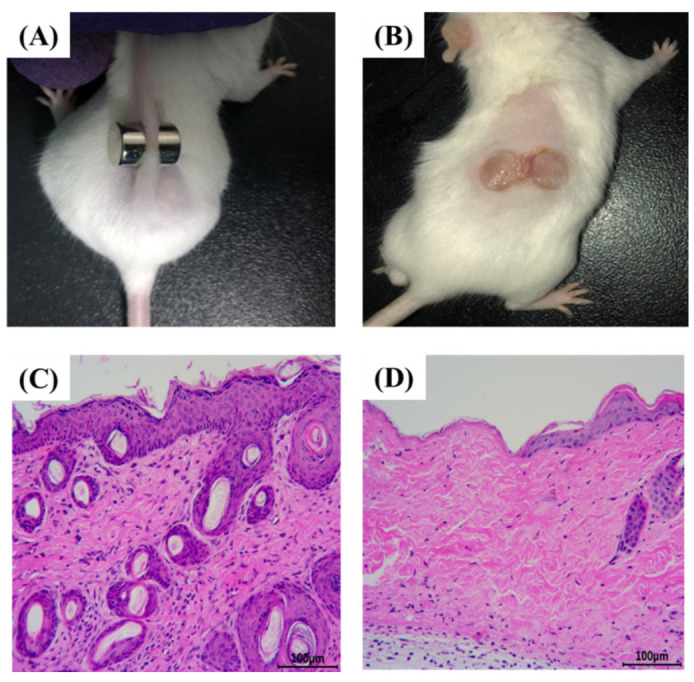
Establishment of PU in mice (*n* = 3). (**A**) A pair of sterile circular magnets was used to establish the PU in mice. (**B**) All mice developed two ulcers after two I/R cycles. (**C**) The H&E staining of normal skin of mice (200×). (**D**) The H&E staining of PU model skin of mice (200×).

Long-term compression of tissues, resulting in tissue ischemia and deformation, causes PU [33,34]. When the pressure on ischemic tissue is relieved, reperfusion of the ischemic tissue blood results in I/R damage [35,36]. In clinical practice, the repeated I/R cycle is considered to be the primary factor leading to the formation of PU as well as one of the most important early PU mechanisms [37,38]. In the current study, murine PU was created using a pair of sterile circular magnets (Figure 1A), and after two I/R cycles, all mice developed two ulcers (Figure 1B). As shown in Figure 1B, the back skin of the mice developed edema or superficial ulceration, where the epidermis or dermis of the skin was broken, and symmetrical wounds were formed, indicating that the early model of PU in mice (stage 2) was successfully constructed. In addition, H&E staining was used to observe pathological changes in both normal and stress-injured mouse skin in order to validate the success of the modeling. After two I/R cycles, compared to the normal murine skin (Figure 1C), the epidermis on the left side of the incision edge of the murine PU skin disappeared, and there was a small amount of exudation on the dermis, indicating that the PU model (phase 2) was successfully constructed (Figure 1D).

### 2.2. Determination of the Rate of Wound Healing

**Figure 2:**
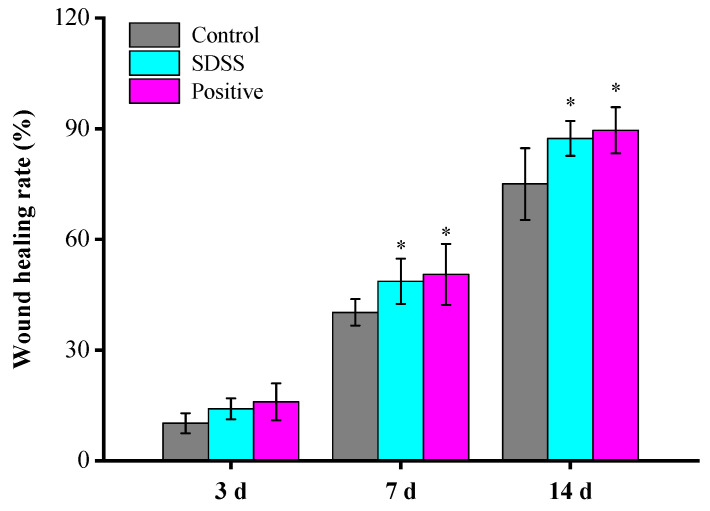
The wound-healing rate of mice (*n* = 5). * *p* < 0.05 in comparison to the control group.

As shown in Figure 2, after 3 days of treatment, there was no statistically significant difference (*p* > 0.05) in the rate of wound healing between the groups. After 7 and 14 days of treatment, the wound-healing rates of the SDSS and positive groups were significantly higher than those of the control group (*p* < 0.05), with no significant difference between the SDSS and positive groups (*p* > 0.05). These findings suggested that SDSS cream could promote the healing of PU in mice.

### 2.3. Histopathological Changes in Mice Skin

**Figure 3:**
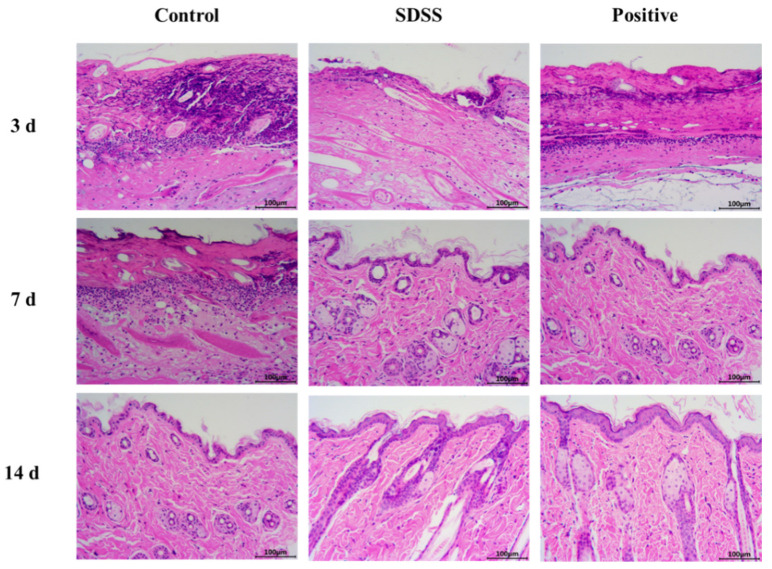
The H&E staining of PU on days 3, 7, and 14 after different treatments (200×).

In the control group, no epidermal cells formed at the PU site of the skin after 3 days of intervention (Figure 3). In the dermis, there were numerous inflammatory cells and necrotic tissue; in the dermis, collagen fibers formed; and in the subcutaneous tissues, there were numerous inflammatory cells. At the edge of the PU in the SDSS group, epidermal cells began to form, and the blood scab began to fall off. Few inflammatory cells were present in the dermis and collagen fibers. In addition, numerous regenerated hair follicles and roots were visible along with fibroblasts and small blood vessels. In the positive group, no epidermal cells formed at the PU site of mice skin; however, inflammatory cells and necrotic tissue remained visible in the dermis, and collagen fibers formed in the dermis. It was possible to see regenerated hair follicles and blood vessels as well as numerous inflammatory cells in the subcutaneous tissues.

In the control group, after 7 days of intervention (Figure 3), there were more inflammatory cells and collagen fibers in the dermis, and there was no formation of epidermal cells at the PU site of mice skin. There were visible signs of regenerating hair follicles and small blood vessels. At the site of PU in the SDSS group, the epidermal cells of the second to the third layer were observed. Few inflammatory cells were observed in the dermis, but there were numerous collagen fibers, fibroblasts, and small blood vessels. The formation of sebaceous glands, hair follicles, and sweat gland ducts was observed. In the positive group, two to three layers of epidermal cells began to form at the site of the PU. In the dermis, there were few inflammatory cells, but numerous collagen fibers, fibroblasts, sebaceous glands, and hair follicles formed. Visible were the vertical trichome muscle and sweat gland ducts.

After 14 days of intervention (Figure 3), the first and second layers of epidermal cells were visible at the PU site of mice skin in the control group. In the dermis, there were few inflammatory cells. There were numerous collagen fibers, fibroblasts, small blood vessels, sebaceous glands, and hair follicles, as well as sweat gland ducts. The basal layer, spinous layer, granular layer, transparent layer, and thin cuticle layer were slightly less prevalent in the SDSS group compared to the positive group at the PU site of mice skin. In the dermis, there were numerous collagen fibers, fibroblasts, and small blood vessels. In addition, there were more sebaceous cells, hair follicle openings in the epidermis, and visible sweat glands and sweat gland ducts. In the positive group, the epidermal cells at the site of the PU formed the basal layer, spinous layer, granular layer, and hyaline layer, and a thin cuticle was visible. In the dermis, there were numerous collagen fibers, fibroblasts, and small blood vessels. There were more sebaceous cells, hair follicle openings in the epidermis, and visible sweat glands and sweat gland ducts. Consistent with our previous studies [32], the above results indicated that SDSS could effectively promote wound healing in skin PU.

### 2.4. Determination of Serum Inflammatory Factors

**Figure 4:**
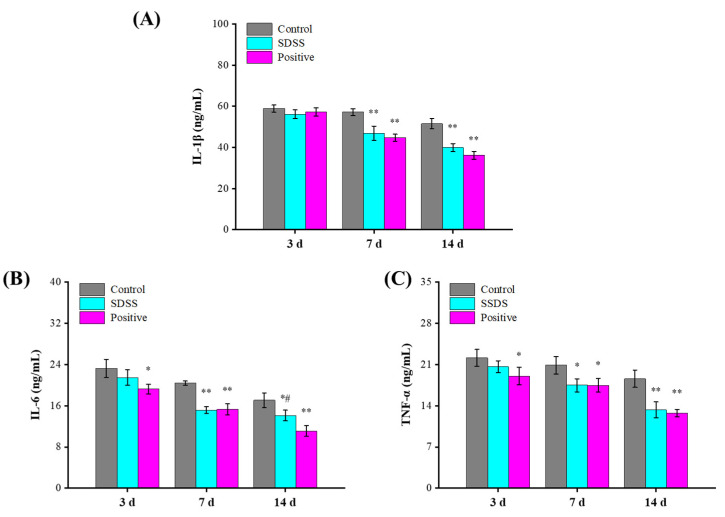
Effect of SDSS on serum levels of inflammatory factors (*n* = 5). (**A**) IL-1β level; (**B**) IL-6 level; (**C**) TNF-α level. * *p* < 0.05, ** *p* < 0.01 when compared to the control group. ^#^ *p* < 0.05 when compared to the positive group.

IL-1β is one of the most common proinflammatory cytokines, and as an initial factor regulating inflammation, it is considered as the most classical inflammatory regulator [39]. As a multifunctional inflammatory factor, IL-1β is crucial for fever, wound healing, inflammatory stimulation, hematopoiesis, immune response, and other physiological responses [40]. IL-6 can activate acute response proteins, play a proinflammatory role, affect the growth of fibroblasts and endothelial cells, and activate the local and systemic defense mechanisms of the host [41]. TNF-α can activate lymphocytes and neutrophils, stimulate vascular endothelial cells to regulate cell metabolism in vivo, and induce tissue cytokine release [42]. In this study, the serum levels of IL-1β, IL-6, and TNF-α were measured to assess the reparative effect of SDSS cream. As shown in Figure 4, after 3 days of intervention, the serum concentrations of IL-1β, IL-6, and TNF-α were lower in the SDSS and positive groups compared to the control group, but this difference was not statistically significant (*p* > 0.05). After 7 and 14 days of intervention, the serum levels of IL-1β, IL-6, and TNF-α in the SDSS and positive groups were significantly lower than in the control group (*p* < 0.05). Zhang et al. [32] demonstrated that SDSS can effectively reduce the TNF-α levels in stage 2 pressure injury wounds in rat models of I/R injury, which is consistent with our findings. Our findings revealed that SDSS reduced the levels of IL-1β, IL-6, and TNF-α, thereby accelerating the healing of PU in mice.

### 2.5. Determination of Antioxidant Indices and MDA Levels

**Figure 5:**
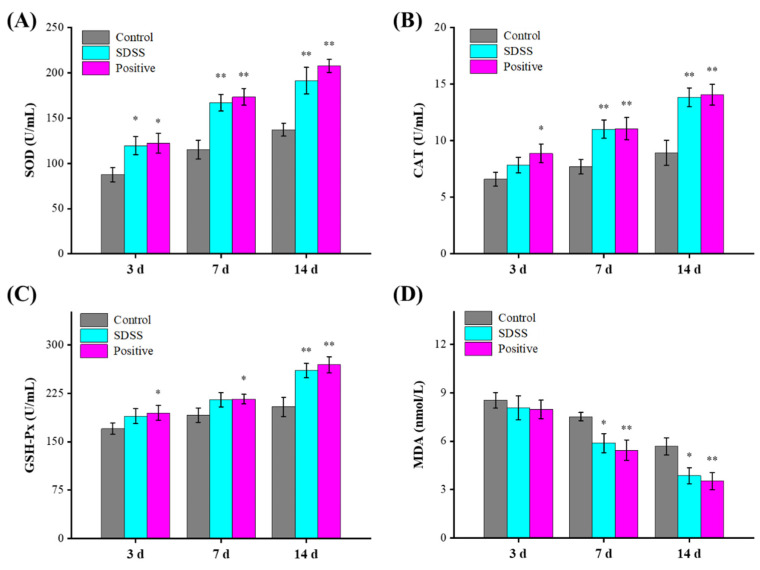
Effect of SDSS on serum SOD (**A**), CAT (**B**), GSH-Px (**C**) activities, and MDA (**D**) levels (*n* = 5). * *p* < 0.05, ** *p* < 0.01 when compared to the control group.

Free radicals contribute significantly to I/R damage [43,44]. The components of free radicals are reactive oxygen species (ROS) and nitroxide radicals (RNS) [45]. Under normal conditions, endogenous free radical scavengers eliminate ROS, thereby rendering endogenous free radicals non-toxic to cells. However, when tissues, organs, and cells experience ischemia and hypoxia, the equilibrium between ROS clearance and ROS production is disrupted [46,47]. When blood and oxygen are restored, an abundance of reactive oxygen species (ROS) is produced and rapidly accumulated [47]. A large number of oxygen free radicals can cause a lipid peroxidation reaction with unsaturated fatty acids in biofilms, leading to their degradation and an increase in the permeability of the cell membrane and organelle membrane, thereby causing damage to the structure and function of the cell [48]. Aside from that, MDA is a secondary metabolite of lipid peroxidation in the cell membrane that can indicate the level of ROS activity in the cell membrane [49]. As shown in Figure 5, the SOD activities of the SDSS and positive groups were significantly higher than those of the control group after 3 days of intervention (*p* < 0.05). The CAT and GSH-Px activities were greater, and the MDA levels were lower compared to the control group, but these differences were not statistically significant (*p* > 0.05). After 14 days of intervention, the serum SOD, CAT, and GSH-Px activities in the SDSS and positive groups were significantly increased (*p* < 0.01), whereas the MDA levels were significantly decreased (*p* < 0.05), indicating that SDSS cream could effectively increase the antioxidant enzymes and decrease the lipid peroxidation to promote the healing of PU in mice.

### 2.6. Effect of SDSS on the Protein Expressions of the Nrf2/HO-1 Pathway

**Figure 6:**
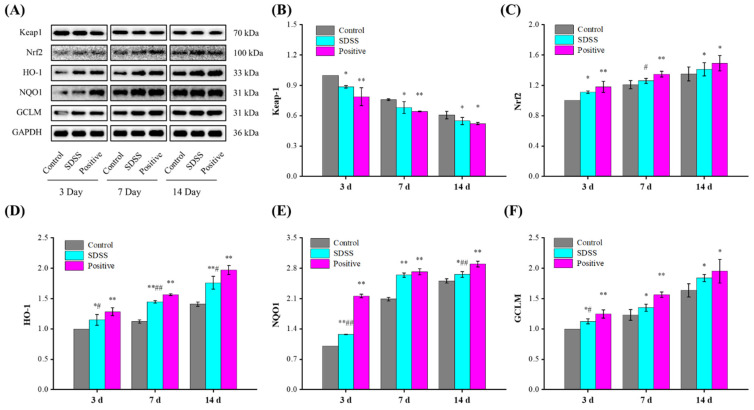
Effect of SDSS on the protein levels of the Nrf2/HO-1 pathway (*n* = 5). (**A**) Western blot; the protein expression levels of Keap1 (**B**), Nrf2 (**C**), HO-1 (**D**), NQO1 (**E**), and GCLM (**F**) levels. * *p* < 0.05, ** *p* < 0.01 when compared to the control group. ^#^ *p* < 0.05, ^##^ *p* < 0.01 when compared to the positive group.

The Nrf2/HO-1 pathway is a multi-organ protective chain responsible for regulating various stress environments [50,51]. Activation of the Nrf2/HO-1 pathway is one of the main mechanisms of cellular defense against oxidative stress, enhancing the coupling reaction and the expression of related antioxidant enzymes (such as SOD, GSH-Px, etc.) [52,53]. Prior studies [50,51] have demonstrated that the Nrf2/HO-1 pathway plays a crucial role in the regulation of PU. The protein expression levels associated with the Nrf2/HO-1 pathway were investigated in this study. As shown in Figure 6, after 3, 7, and 14 days of intervention, the protein expression levels of Keap1 decreased significantly, while the protein expression levels of Nrf2, HO-1, NQO1, and GCLM increased significantly in the SDSS and positive groups relative to the control (*p* < 0.05 or *p* < 0.01). These results suggested that the protective mechanism of SDSS may be related to its activation of the Nrf2/HO-1 pathway.

### 2.7. Effect of SDSS on the Protein Expressions of the NF-κB Pathway

**Figure 7:**
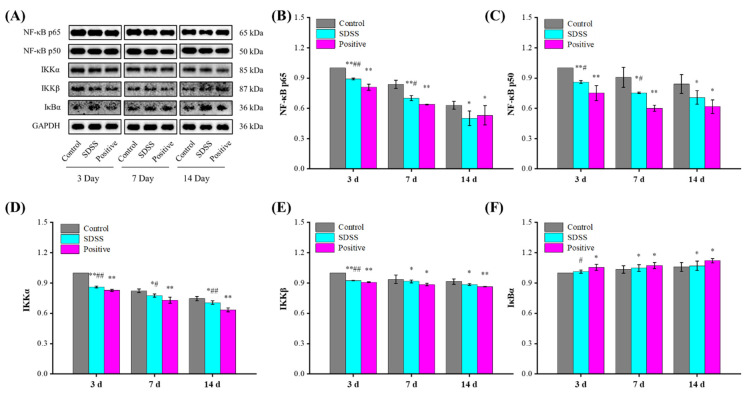
Effect of SDSS on the protein levels of the NF-κB pathway (*n* = 5). (**A**) Western blot; the protein expression levels of NF-κB p65 (**B**), NF-κB p50 (**C**), IKKα (**D**), IKKβ (**E**), and IκBα (**F**) levels. * *p* < 0.05, ** *p* < 0.01 when compared to the control group. ^#^ *p* < 0.05, ^##^ *p* < 0.01 when compared to the positive group.

In addition, the NF-κB pathway plays a crucial role in the regulation of PU [50,54]. In response to a variety of mechanical stresses, cytokines, or chemical signals, NF-κB protein expression is increased, resulting in the phosphorylation of IB kinase and subsequent degradation of IκBα protein. With the degradation of IκBα protein, NF-κB dissociates from the NF-κB-IκBα complex and translocates to the nucleus, initiating the transcription of factors associated with a variety of biological events, such as inflammatory factors [55]. As shown in Figure 7, after 7 and 14 days of intervention, the protein expression levels of NF-κB p65, NF-κB p50, IKKα, and IKKβ were significantly decreased, whereas the IκBα levels were significantly increased in the SDSS and positive groups compared with the control group (*p* < 0.05 or *p* < 0.01). Combined with the results of serum inflammatory factors, our findings suggested that SDSS could inhibit the NF-κB pathway to reduce PU-induced inflammation.

## 3. Materials and Methods

### 3.1. Materials and Reagents

Sodium Danshensu (SDSS) was purchased from Aladdin (Shanghai, China). Mucopolysaccharide-polysulfate cream (Hirudoid^®^) was provided by Hangzhou Women’s Hospital (Hangzhou, China). Enzyme-linked immunosorbent assay (ELISA) kits for interleukin (IL)-6, IL-1β, and tumor necrosis factor (TNF)-α were purchased from Boster (Wuhan, China). The malondialdehyde (MDA), superoxide dismutase (SOD), glutathione peroxidase (GSH-Px), catalase (CAT), and hematoxylin–eosin (H&E) staining kits were purchased from Jiancheng (Nanjing, China). The primary antibodies against NF-κB p65 (AF0246), NF-κB p50 (AF1246), IKKα (AF0198), IKKβ (AF7200), and IκB-α (AI096) were purchased from Beyotime (Shanghai, China). The primary antibodies against Keap1 (BF0010), Nrf2 (AF0639), HO-1 (AF5393), GCLM (DF7268), and NQO1 (DF6437) were purchased from Affinity Biosciences (Liyang, China). The primary antibody against GAPDH (K200057M) was purchased from Solarbio (Beijing, China).

### 3.2. Preparation of SDSS Cream

Under aseptic conditions, the SDSS cream was prepared by melting the water phase, which contained 30 mg SDSS, 1 g glycerol, 0.05 g triethanolamine, and 7.42 g water, at 80 °C. Then, while stirring, the water phase was added slowly to the oil phase, which contained 0.5 g stearic acid, 0.5 g vaseline, and 0.5 g liquid paraffin. After adding them, quickly stir them in a water bath (80 °C) for 3 min, and then continue stirring at room temperature until condensation occurs. Thus, 10 g of a cream containing 0.03% SDSS (*w*/*w*) was prepared. 

### 3.3. Animal Experiments

Male ICR mice (18–22 g, 4–6 weeks) were purchased from the Zhejiang Academy of Medical Sciences. After a week of adaptive feeding, all mice received an intraperitoneal injection of 2% sodium pentobarbital to induce anesthesia. The hair on the backs of the mice was then shaved and removed with 6% sodium sulfide using scissors. Afterwards, 70% ethanol was used to clean and disinfect the area to be shaved. In accordance with Stadler’s [56] mice modeling, a pair of sterile circular magnets (10 mm in diameter, 5 mm in thickness, and 2.8 g in weight) were used to clamp approximately 5 mm of skin on the hair removal area of mice. A single I/R cycle consists of 12 h of magnet placement (ischemia period) followed by 12 h of magnet removal (reperfusion period). After 2–3 I/R cycles, each mouse developed two ulcers (Figure 1).

The mice were then divided at random into 3 groups (*n* = 15 per group). In the control group, mice with skin wounds were treated with 0.5 g of cream without SDSS. The wounds of mice in the treatment group (SDSS) were treated with 0.5 g of cream, while those in the positive control group were treated with 0.5 g of Hirudoid^®^ cream (positive). The drug was administered daily for 14 days. To evaluate the wound closure of SDSS, mice were anesthetized and sacrificed 3, 7, and 14 days later (*n* = 5 for each time point) by cervical dislocation.

### 3.4. Determination of the Rate of Wound Healing

On days 0, 3, 7, and 14, the wound-healing pattern was photographed with a digital camera, and the wound area was calculated using Image J software. The rate of wound closure (%) was computed as follows [57]:

Wound closure rate (%) = (A_0_ − A_t_)/A_0_ × 100%.

where A_0_ is the wound area at 0 days, and A_t_ is the wound area at a particular day.

### 3.5. Histological Analysis

The tissues of the skin were fixed with 4% paraformaldehyde and embedded in paraffin. The tissues were then sectioned to a thickness of 5 m. The tissue slides were stained with H&E in order to evaluate the effect of SDSS on wound repair. The micrographs were taken with a CX31 light microscope (Olympus, Tokyo, Japan).

### 3.6. Biochemical Analysis

Eyeball extirpating was used to obtain blood samples from mice, and serum was extracted from fresh blood by centrifugation (6000× *g*, 4 °C, 3 min). The serum levels of IL-6, IL-1β, and TNF-α were measured according to Boster’s instructions. The serum MDA concentration and SOD, GSH-Px, and CAT activities were measured according to Jiancheng’s instructions.

### 3.7. Western Blot Analysis

The PU skin sites were used for Western blotting, and the Western blotting was performed in accordance with previous research [58]. Each sample underwent SDS-PAGE electrophoresis with 30 g of protein; the protein bands were then transferred to a PVDF membrane and sealed for 1 h with 5% bovine serum albumin (BSA). At 4 °C overnight, primary antibodies (GAPDH, Nrf2, HO-1, GCLM, NOQ1, NF-κB P65, NF-κB P50, IKKα, IKKβ, and IκBα) were added and incubated. The AlphaView software was used to obtain and quantify the protein bands of interest (version 3.4.0, ProteinSimple, San Jose, CA, USA).

### 3.8. Statistical Analysis

All results were analyzed using SPSS 26.0 software and expressed as the mean standard deviation. The one-way ANOVA and Duncan’s test were conducted, and a *p* < 0.05 was deemed statistically significant.

## 4. Conclusions

**Figure 8:**
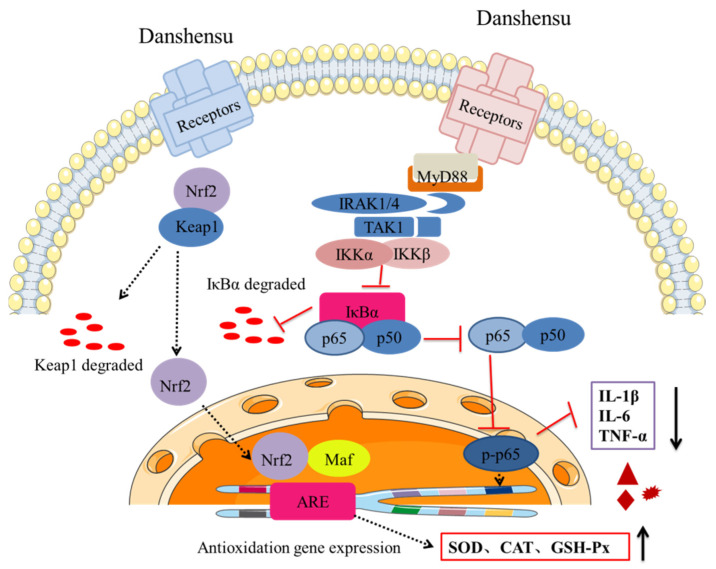
SDSS cream repaired PU in mice, possibly by activating Nrf2/HO-1 pathway and inhibiting the NF-κB pathway.

In conclusion, SDSS cream could promote the wound-healing rate of PU in mice effectively. The H&E staining results demonstrated that the histomorphology of mice with PU was significantly enhanced by SDSS. After treatment with SDSS cream, the serum levels of IL-1β, IL-6, and TNF-α decreased significantly, while the serum SOD, CAT, and GSH-Px activities increased significantly. In addition, our findings suggested that the protective mechanism of SDSS may be associated with its activation of the Nrf2/HO-1 pathway and inhibition of the NF-κB pathway (Figure 8). However, the optimal dosage of SDSS was not systematically investigated in this study; in the future, more animal experiments can be conducted to determine the optimal dosage of the drug. In addition, transcriptomics and proteomics can be used to further investigate the mechanism of SDSS in order to clarify how it promotes wound healing in mice with PU.

## Data Availability

Data is contained within the article.
